# Can we predict the postoperative infections?

**DOI:** 10.1186/cc11989

**Published:** 2013-03-19

**Authors:** T Mózes, K Gornicsar, A Grosz, ZS Domján, I Buzogány

**Affiliations:** 1 Ministry of Defense Health Centre, Budapest, Hungary; 2 Péterfy Hospital, Budapest, Hungary; 3 University of Szeged, Hungary

## Introduction

Many patients develop infections following operations. Decreased immune competence has been demonstrated in acute neurological conditions. A strong cytokine-mediated anti-inflammatory response was observed in stroke patients at infection, although infection due to the decreased proinflammatory mediators can be expected as well. To investigate this question the following experiment was performed.

## Methods

Twenty-two urinary bladder cancer patients with radical cystectomy and lymphadenectomy were studied. Blood samples were taken on day 0 (before) and days 1, 3, 6, 9 and 14 after operation as well as on days 30, 60, 90 and 270 during follow-up. TNFα, soluble TNFα receptor I and IL-6 levels in sera were determined by HS ELISA and/or ELISA. Plasma ACTH and cortisol values were measured by RIA kits.

## Results

From 22 patients, eight deep wound and urine infections were found in 14 days and six urine and wound infections in 30 days after surgery, all survived. All patients were bacterially contaminated, as wound samples taken at the end of operation demonstrated. On day 0 the circulating TNFα values were lower in infected patients. TNF started to increase from day 3 to day 9, never reaching values of the uneventful healing group. Soluble TNF receptor I, IL-6, ACTH, and cortisol concentrations did not demonstrate any difference on day 0 but from day 1 started to increase transiently, reaching higher levels in septic patients.

## Conclusion

A low proinflammatory response is a key facilitating factor for the development of infection. Measuring serum TNFα levels before and after operations can thus predict the outcome.

